# Valosin-containing protein (VCP/p97) inhibition reduces viral clearance and induces toxicity associated with muscular damage

**DOI:** 10.1038/s41419-022-05461-w

**Published:** 2022-12-01

**Authors:** Marta del Rio Oliva, Michael Basler

**Affiliations:** 1 Division of Immunology, Department of Biology, University of Konstanz, Konstanz, Germany; 2 Biotechnology Institute Thurgau at the University of Konstanz, Kreuzlingen, Switzerland

**Keywords:** Cell death and immune response, Preclinical research, Infection

## Abstract

Valosin-containing protein (VCP)/p97 has emerged as a central regulator of the ubiquitin–proteasome system by connecting ubiquitylation and degradation. The development of CB-5083, an ATPase D2-domain-selective and orally bioavailable inhibitor of VCP/p97, allows targeting of the ubiquitin–proteasome system in human diseases. In this study, we evaluated the effect of CB-5083 on the immune response in mice by using the lymphocytic choriomeningitis virus (LCMV) as an infection model. We demonstrate that LCMV infection increased the susceptibility to CB-5083 treatment in a CD8-independent manner. Administration of CB-5083 to mice reduced the cytotoxic T cell response and impaired viral clearance. Compared to uninfected cells, CB-5083 treatment enhanced the unfolded protein response in LCMV-infected cells. Administration of CB-5083 during the expansion of CD8^+^ T cells led to strong toxicity in mice within hours, which resulted in enhanced IL-6 levels in the serum and accumulation of poly-ubiquitinated proteins. Furthermore, we linked the observed toxicity to the specific formation of aggregates in the skeletal muscle tissue and the upregulation of both lactate dehydrogenase and creatine kinase in the serum.

## Introduction

The AAA-ATPase p97, also called valosin-containing protein (VCP), is a hexameric protein that uses the energy of ATP hydrolysis to structurally remodel client molecules, thereby regulating a wide range of processes [1]. Its main role relies on its capability to bind directly—or indirectly—to ubiquitinated substrates, which facilitates steps downstream of ubiquitylation such as degradation by the proteasome [2]. Interestingly, a population of proteasomes has been observed in proximity to the ER translocon [3], in which VCP/p97 is required to control the passage of substrates. VCP/p97 has not only been suggested to be essential in the retrotranslocation of substrates out of the ER [4] but it also interacts with the 19S of the 26S proteasome, acting as an unfoldase protein and replacing the function of the proteasomal resident AAA proteins [5]. VCP/p97 contains an N-terminal domain and two ATPase domains (D1 and D2) which form two stacked rings [6]. The energy released through ATP hydrolysis is most likely converted into a mechanical force that disassembles protein complexes, partially unfolds substrate proteins, or segregates substrates from membranes and other cellular structures [7]. Depending on associated co-factors [8] VCP/p97 has a wide range of functions, including ER-associated degradation (ERAD), mitochondrial-associated protein degradation [9, 10], the ubiquitin–proteasome system (UPS) [11], DNA replication, and break repair [12, 13] as well as NF-κB activation [14, 15]. Missense mutations of VCP/p97 lead to the autosomal dominant disease named “inclusion body myopathy with early-onset Paget disease and frontotemporal dementia” (IBMPFD) [16]. This disease is characterized by progressive myopathy with abnormal bone homeostasis [17].

Through the years, several VCP/p97 inhibitors have been developed [18]. CB-5083 is a potent, selective, and orally bioavailable inhibitor of VCP/p97, which displays properties suitable for clinical trials [19, 20]. In vitro treatment of tumor cells with CB-5083 led to the retention of ERAD substrates, accumulation of C/EBP homologous protein (CHOP), and K48 poly-ubiquitinated proteins in addition to p62 reduction [20]. Furthermore, CB-5083 treatment demonstrated the induction of apoptosis and antitumor activity in xenograft models [20] as well as its effectiveness in various multiple myeloma models [21].

The key role of VCP/p97 in host cell function augurs its importance for virus infection. Indeed, VCP/p97 has been demonstrated to participate in numerous stages of viral infection [22–24]. Despite its impact on viral homeostasis, little is known of VCP/p97’s role during in vivo infection. Therefore, we characterized the immune response of C57BL/6 mice infected with LCMV and treated with CB-5083.

## Material and methods

### Mice, viral infection, and treatments

C57BL/6 mice (H-2^b^) were originally obtained from Charles River Laboratories and further bred in the animal facilities of the University of Konstanz. Mice were kept under pathogen-free conditions with a light-dark cycle of 12 h and used at 8–12 weeks of age, at a weight of 20–25 g, in accordance with the rules of the authority of Regierungspräsidium Freiburg (G-18/10). The number of mice for each set of experiments was determined using G*Power 3.0.10 (comparison of two independent groups, effect size *d*: 0.79, *α* err prob: 0.05 [25]. Food and water were provided ad libitum. Mice were infected with 200 pfu LCMV-WE i.v. CB-5083 was dissolved in 0.5% methyl cellulose in water and administered by oral gavage (50 mg/kg) [19, 20]. Control groups (vehicle) were treated with 0.5% methyl cellulose in water. Mice were randomly allocated to experimental groups and no blinding method was used. There was no animal exclusion criteria. Animal experiments were conducted in full accordance with the ARRIVE guidelines.

### Viral titers

Titers of LCMV in spleens of infected mice were determined on adherent fibroblast cells (MC57) as previously described [26]. Spleens from infected mice were collected and homogenized on ice using a tissue grinder. A 1:10 sample titration was prepared and combined with MC57 cells at 1.6 × 10^5^ cells/ml. The plate was kept in a CO_2_ incubator at 37 °C for 2 h until a monolayer was formed. Then, an overlay 1:1 mixture of 2× DMEM and 2% methylcellulose was added. After 2 days at 37 °C, the cells were fixed and permeabilized with 1% Triton X-100 for 20 min at room temperature. Non-specific binding was blocked with PBS containing 10% FBS. Samples were incubated with rat anti-LCMV-NP Ab (undiluted rat hybridoma supernatant) [27] in PBS/2% FCS for 60 min at room temperature. Then, the cells were washed twice, and further incubated with a peroxidase-conjugated goat anti-rat anti-IgG antibody (Jackson ImmunoResearch Lab, # 112–035–003.) diluted 1:400 for 60 min at room temperature. After two washing steps the samples were incubated with 0.2 M Na_2_HPO_4_, 0.1 M Citric acid, 30% H_2_O_2_ and 20 mg OPD. After 20 min of incubation at room temperature the color reaction buffer was removed and the samples were washed. Viral titer quantification was visually analyzed.

### Immunoblotting

Immunoblotting was performed as described in [28]. The antibodies used were Ubiquitin (FK2, #BML-PW8810–0100), from Enzo Life Science or Bip (C50B12, #3177), IκBα (N-terminal Antigen, L35A5, #4814) and phospho-elF2α (Ser51, #9721) from Cell Signaling. All the antibodies were used in 1:1000 dilution. As secondary antibodies, anti-mouse/rabbit IRDye800 CW or IRDye680RD (#926–32210, #926–32211, 926–68070, #926–68071) (1:10,000) antibodies were used. Signals were quantified with the LI-COR Odyssey Imager and Image Studio Lite Vers. 5.2. The uncropped original immunoblots are depicted in Supplementary Fig. 1.

### Flow cytometry and proteostat® staining

Surface staining was performed with antibodies diluted in FACS-buffer (PBS, 2% FCS, 2 mM NaN_3_, and 2 mM EDTA) for 20 min at 4 °C, followed by three washing steps. Blood samples were incubated with 2 ml FACS lysing solution (BD Bioscience. # 349202) to lyse erythrocytes. The antibodies used were CD11b-PE-Cy7 (M1/70, #25-0112-82), CD19-FITC (eBio1D3, #11-0193-82), CD8-FITC (53-6.7, #MA1-10303), CD4-PE (GK1.5, #12-0041-85), F4/80-PE (BM8, #12-4801-82), NK1.1-APC (PK136, #17-5941-82) from eBiosciences or IFN-γ-FITC (XMG1.2, #562019) from BD Pharmingen. All antibodies were used at a 1:150 dilution in FACS buffer. For proteostat® staining, the organs were collected in 1× PBS and a single cell suspension was prepared by digestion with 1 mg/ml DNAse I (Sigma, #DN25) and 1 mg/ml collagenase D (Roche, #50-100-3282) in HBSS (10 mM Hepes) in a gentleMacs Dissociator (Miltenyi Biotec). Proteostat® staining was performed using an Aggresome detection kit (Enzo®, #ENZ-51035-K100) according to the manufacturer’s protocol. Samples were measured using FacsVerse (BD Biosciences). The gating strategy for flow cytometry is depicted in Supplementary Fig. 2. Flow cytometry data were analyzed with FlowJo v10 (BD Biosciences).

### Intracellular cytokine staining

Intracellular cytokine staining was performed as described previously [29]. Spleens from LCMV-infected mice were collected and a single-cell suspension was prepared using a 70 µm nylon mesh. Cells were stimulated with 2 × 10^-6^ M of the corresponding peptides GP_33–41_, NP_276–396_, NP_396–404_, NP_205–212_, and GP_118–126_ (peptides & elephants). Plates were incubated for 6 h at 37 °C. Then, intracellular staining for IFN-γ producing CD8^+^ cells was performed. The staining, fixation, and permeabilization of the cells were performed exactly as previously detailed [30]. 4% paraformaldehyde (in PBS) for 5 min at 4 °C was used to fix the cells. The samples were subsequently permeabilized with Perm-buffer (PBS, 0.1% saponin, 2% FCS, 2 mM NaN_3_, and 2 mM EDTA) and stained overnight with IFN-γ-FITC (XMG1.2 BD Pharmingen, #562019) (1:150). After three washing steps, samples were measured using the FacsVerse (BD Biosciences).

### ELISA and serum analysis

Whole blood was collected via cardiac puncture and serum was obtained after centrifugation at 10,000 × *g* for 5 min at 4 °C. ELISA for IFN-γ (#88-7314-86), IL-6 (#88-7064-88), IL-1β (#88-7013-88), and TNF-α (#88-7324-88) (all from ThermoFisher Scientific) was performed according to the manufacturer’s protocol. Shortly, 96-well plates were coated overnight at 4 °C with the corresponding capture antibody. Then, they were washed and blocked with assay diluent for one hour. After washing, both samples and standard dilutions were incubated for 2 h at room temperature. Then, samples were washed and incubated with detection antibody and Avidin-HRP in assay diluent for 1 h at RT. Substrate solution was added until an appropriate blue color was produced. The reaction was stopped by adding 1 M H_2_SO_4_. The absorbance was measured using an absorbance reader. Serum samples were diluted 1:3 in dilution buffer. The measurement of myoglobin concentration in the serum was performed with the Mouse Myoglobin ELISA Kit (Abcam, #ab210965). The activity assays for the determination of the creatine kinase and lactate dehydrogenase were performed using the Creatine Kinase Activity Assay Kit (Abcam, #ab155901) and Lactate Dehydrogenase Activity Assay Kit (Abcam, #ab282925).

### Analysis of the unfolded protein response

MC57 cells (kind gift of Maries van den Broek, University of Zurich; not tested for mycoplasma) were infected with LCMV-WE (MOI 0.1) for 1 h. Afterward, cells were washed and incubated with 5% MEM for 24, 36, or 48 h at 37 °C. Six hours before harvesting, the cells were treated with 5 µM CB-5083 or DMSO at 37 °C. As controls, samples were treated with 4 µg/ml of tunicamycin. RNA was isolated from the pellets using the RNeasy Mini kit (Qiagen, #74106). After purity assessment with a NanoVue (GE Healthcare), cDNA was synthesized using Biozym cDNA synthesis kit (#331470 L). Real-time RT-PCR (Biozym Blue S′Green Kit, #331416XL) was performed in a Biometra TProfessional Thermocycler (Analytik Jena). Primers used were ATF4: 5′-GGGTTCTGTCTTCCACTCCA-3′ and 5′-AAGCAGCAGAGTCAGGCTTTC-3′ [31], ATF6: 5′-GGACGAGGTGGTGTCAGAG-3′ 5′-GACAGCTCTTCGCTTTGGAC-3′ [32], BCL2: 5′-TGAGTACCTGAACCGGCATCT-3′ 5′-GCATCCCAGCCTCCGTTAT-3′ [33] and XBP1S: 5′-CTGAGTCCGAATCAGGTGCAG-3′ 5′-GTCCATGGGAAGATGTTCTGG-3′ [34]. Relative gene expression was normalized to hypoxanthine-guanine phosphoribosyltransferase (Hprt) (5′-CCAGCAGGTCAGCAAAGAACTTA-3′ 5′-TGGACAGGACTGAAAGACTTG-3′ [35].

### IκBα kinetics

2 × 10^6^ MC57 cells were treated with 5 µM CB-5083 or DMSO for 6 h. Then, cells were stimulated with 200 U/ml TNF-α. Cells were harvested after 5, 15, 30, 45, 60, or 120 min. Cells were centrifuged, washed, and lysed in lysis buffer (1% (w/v) Triton, 10 mM Trizma base, 150 mM NaCl, pH 6.8) and analyzed by western blot.

### Statistical analysis

Statistical analysis was performed using Prism 9.1 (Graphpad). Results are expressed as scattered individual values and mean ± S.D. Shapiro–Wilk (W) test was used to analyze the normal distribution of the variables (*p* > 0.05). Quantitative data without a normal distribution were analyzed with non-parametric tests (Kruskal–Wallis or Mann–Whitney test), and data with a normal distribution were analyzed with parametric tests (unpaired *t* test, ordinary one-way or two-way ANOVA). No statistical methods were used to predetermine the sample size. Statistical significance was achieved when *p* < 0.05; *p < 0.05, **p < 0.01, ***p < 0.001, and ****p < 0.0001. The variance was similar between the groups that were being statistically compared.

## Results

### CB-5083 increases the susceptibility to LCMV infection

**Fig. 1:**
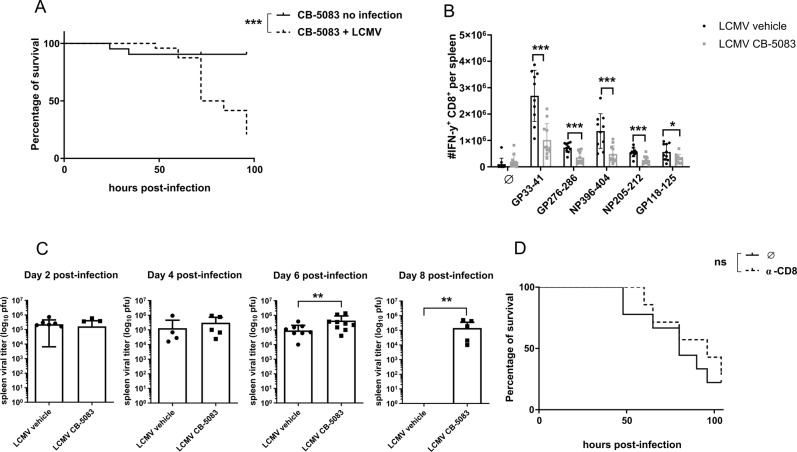
CB-5083 increases the susceptibility to LCMV infection. **A** C57BL/6 mice were infected with LCMV (*n* = 10), or left uninfected (*n* = 10), and treated daily with 50 mg/kg CB-5083. The percentage of survival is shown. Data were pooled from two independent experiments and analyzed by Gehan–Breslow–Wilcoxon test. **B** C57BL/6 mice were infected with LCMV and treated with 50 mg/kg CB-5083 (*n* = 11) or vehicle (*n* = 10) on d0 and d2 post-infection. Spleens were harvested on d8 post-infection, left unstimulated (∅) or stimulated in vitro with the indicated LCMV peptides for 5 h, and analyzed by flow cytometry after staining for CD8 and intracellular IFN-γ. The absolute cell number of IFN-γ-producing CD8^+^ cells ± SD is shown on the *γ*-axis. The CTL response to each peptide (pooled from two independent experiments) was statistically analyzed by unpaired *t* test. **C** Mice were infected on d0 and treated with CB-5083 or vehicle on d0 and d2 post-infection. Viral titers of LCMV in the spleen were analyzed on indicated days post LCMV infection. Viral titers are presented as the mean ± SD of 8–11 mice (pooled from 2 independent experiments). For each day, a Mann–Whitney *t* test was performed. **D** Mice were injected i.p. with a CD8 depleting antibody (*n* = 7) or left untreated (∅) (on d-3). Three days later (d0), they were infected with LCMV and treated daily with 50 mg/kg CB-5083. α-CD8 was re-administered on d2 post-infection. On the *γ*-axis, the percentage of survival is depicted. Data pooled from two independent experiments were analyzed by the Gehan–Breslow–Wilcoxon test. All values represent mean ± SD. ns = non significant, *p < 0.05, **p < 0.01, and ***p < 0.001.

To verify the function of VCP/p97 as a proteasome-upstream element, we analyzed ubiquitylation in mouse B8 fibroblasts treated with increasing concentrations of CB-5083. We observed a strong increase in the poly-ubiquitinated protein pool (Supplementary Fig. 3), confirming previous results [21]. To characterize the effect of VCP/p97 inhibition on the anti-viral immune response, we infected C57BL/6 mice with LCMV-WE. CB-5083 was administered by oral gavage daily at 50 mg/kg body weight, a dose which results in 62% tumor growth inhibition in vivo [20]. Both infected and uninfected mice were treated daily with CB-5083 and survival of mice was analyzed. Uninfected mice displayed a minor disturbance in their survival (Fig. 1A), which was visible around 38 h post-infection. Furthermore, in uninfected mice CB-5083 treatment had no influence on the health status. On the other hand, LCMV-infected mice treated daily with CB-5083 displayed a massive disruption in their health status, peaking at approximately 70 h post-infection, a time point at which more than 50% of the mice had to be euthanized (Fig. 1A).

LCMV infection in mice induces a strong cytotoxic T cell (CTL) response, which peaks at around d8 post-infection [36]. To investigate the effect of VCP/p97 inhibition on the CTL response, the reaction to five LCMV-derived CD8 epitopes was assayed in CB-5083 or vehicle-treated mice by intracellular cytokine staining (ICS) for IFN-γ (Fig. 1B). The CTL response to all analyzed epitopes was significantly reduced upon CB-5083 treatment, which reflects the inability of the mice to cope with the viral infection in presence of the VCP/p97 inhibitor (Fig. 1A). Indeed, in contrast to vehicle-treated mice, LCMV could not be cleared within 8-days after infection in the spleen of CB-5083-treated mice (Fig. 1C). Interestingly, VCP/p97 inhibition had no effect on the initial viral expansion (d2 and d4), indicating that the innate immune response, which is responsible to control viral expansion in the first days post-infection, is not grossly affected by VCP/p97 inhibition.

The reduced CTL response (Fig. 1B) and the failure to clear LCMV (Fig. 1C) in CB-5083-treated LCMV-infected mice indicated that the CD8^+^ cells might be responsible for CB-5083 toxicity in LCMV-infected mice (Fig. 1A). To investigate this, CD8^+^ T cells were depleted prior to LCMV infection. Depletion of CD8^+^ cells in blood was approximately 95% (Supplementary Fig. 4). No difference in survival rate could be observed in LCMV-infected CB-5083-treated mice in the presence or absence of CD8^+^ cells (Fig. 1D), indicating that CB-5083 susceptibility after LCMV infection is independent of CD8^+^ cells.

**Fig. 2:**
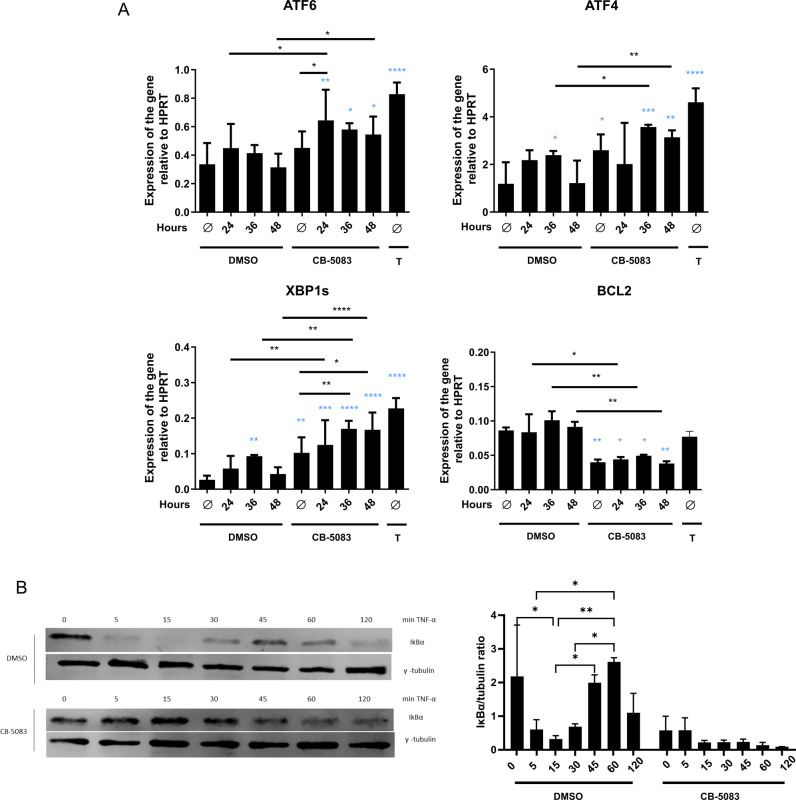
Unfolded protein response induction and cytokine-induced degradation of IκBα by CB-5083. **A** MC57 cells were infected with LCMV for 24, 36, and 48 h or left uninfected (Ø). 6 h before harvesting the cells they were treated with 5 µM CB-5083 or DMSO. As a positive control, uninfected cells were treated with 4 µg/ml of tunicamycin (T) for 6 h. The relative expression of the indicated genes analyzed by real-time RT-PCR is shown. The data was normalized to HPRT. A two-way ANOVA followed by a Fisher’s LSD test was performed. The statistical comparison of the data with the uninfected DMSO-treated sample is depicted in blue color. Data were pooled from four independent experiments. **B** MC57 cells were incubated with DMSO or 5 μM CB-5083 for 6 h. Then the cells were stimulated with 200 U/ml TNF-α and harvested at indicated time points. Samples were lysed and an SDS-PAGE and immunoblot for IκBα were performed. γ-tubulin was used as a loading control. The experiment was repeated twice yielding similar results. Data was pooled from two independent experiments and analyzed by a two-way ANOVA followed by Tukey test. All values represent mean ± SD. *p < 0.05, **p < 0.01, ***p < 0.001, and ****p < 0.0001.

In light of the prior data, we hypothesize that treatment with CB-5083 concomitant to LCMV infection could lead to a massive induction of the UPR. The associated cell death could then be responsible for the toxicity observed in the mice. Since viral infection and its relationship to ER homeostasis has barely been investigated, we decided to analyze the effects of VCP/p97 inhibition on the UPR upon viral infection in vitro in mouse fibrosarcoma MC57 cells. Approximately 80% of cells were infected with LCMV 24 h post-infection (Supplementary Fig. 5). Remarkably, no difference in the percentage of infected cells was detected between CB-5083-and DMSO-treated cells, which is congruent with the in vivo experiments at d2 and d4 post-LCMV infection (Fig. 1C). Hence, viral replication seems not to be affected by VCP/p97 inhibition. Real-time RT-PCR analysis was performed to examine the transcriptional fold change of key regulators of the UPR (Fig. 2A). We detected a transient increase of both ATF4 and XBP1s 36 h after LCMV infection. An upregulation of both ATF4 and XBP1s were observed after 6 h of treatment with CB-5083. Interestingly, no change in the gene expression was discovered for the ATF6 branch. However, we noticed that treatment with CB-5083 in presence of LCMV reinforced the upregulation of both ATF6 and XBP1s. No alteration in the transcriptional expression of BCL2 was detected upon viral infection. Furthermore, we discovered that the treatment with CB-5083 downregulated the expression of the anti-apoptotic BCL2 gene in non-infected cells, which was not altered upon LCMV infection. Additionally, we analyzed the degradation of the inhibitor of NF-κB (IκB)α in MC57 cells and confirmed [37] that CB-5083 impeded the TNFα-induced degradation of IκBα (Fig. 2B). Similar results were found in the mouse B8 fibroblasts cell line and in the T1 T cell line (Supplementary Fig. 6). Blocking the activation of NF-κB might contribute to the observed susceptibility of LCMV-infected CB-5083-treated mice.

### VCP/p97 inhibition during LCMV infection alters several immune cell populations in the blood and the spleen

**Fig. 3:**
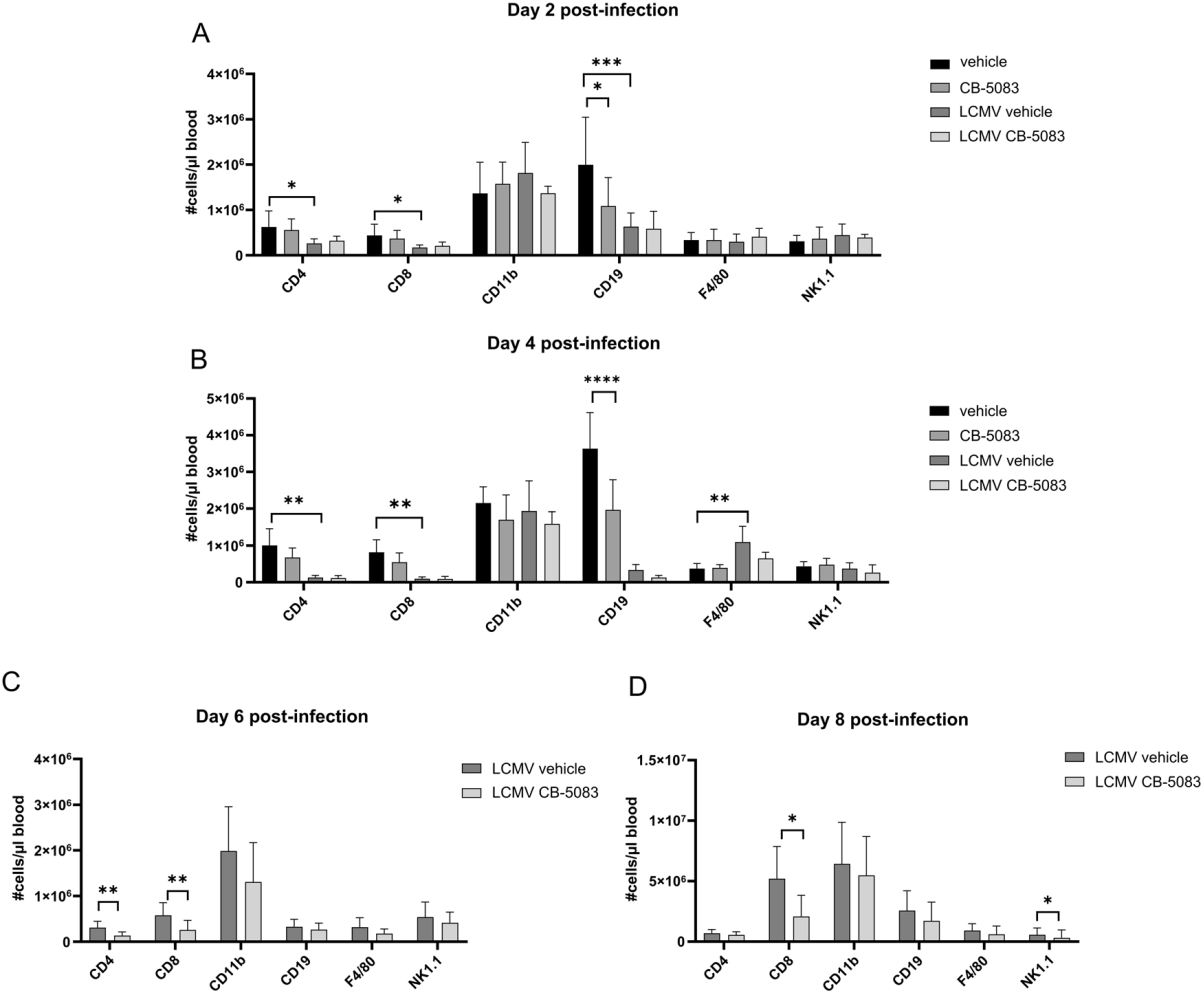
Analysis of immune cells in the blood of LCMV-infected CB-5083-treated mice. C57BL/6 mice were infected with LCMV (d0), or left uninfected (**A**, **B**), and treated with 50 mg/kg CB-5083 or vehicle on d0 and d2 post-infection. Blood samples were collected on d2 (**A**), d4 (**B**), d6 (**C**), and d8 (**D**). Blood cells were stained for CD8, CD4, CD11b, CD19, F4/80, and NK1.1 and analyzed by flow cytometry. On the *γ*-axis, the absolute cell number/µl of blood of each population is shown. Values represent mean ± SD (*n* = 5–11 per group). **A** and **B** were analyzed by individual ordinary one-way ANOVA or Kruskal–Wallis test with multiple corrections performed by Tukey or Dunn’s test, respectively. **C** and **D** were analyzed by unpaired *t* test or Mann–Whitney test. Data was pooled from two independent experiments. All values represent mean ± SD. *p < 0.05, **p < 0.01, ***p < 0.001, and ****p < 0.0001.

**Fig. 4:**
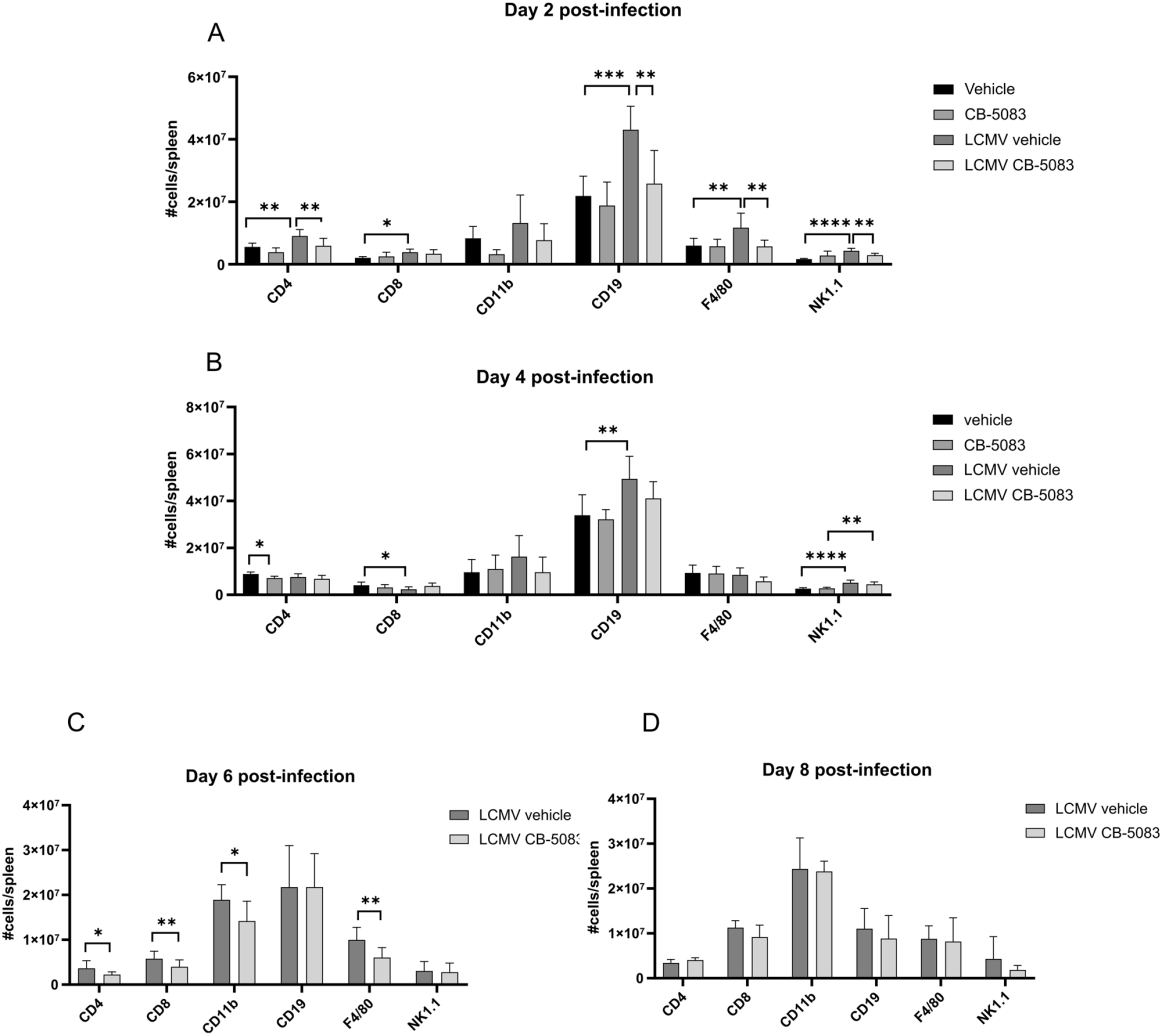
Analysis of immune cells in the spleen of LCMV-infected CB-5083-treated mice. C57BL/6 mice were infected with LCMV (d0), or left uninfected (**A**, **B**), and treated with 50 mg/kg CB-5083 or vehicle on d0 and d2 post-infection. Spleens were collected on d2 (**A**), d4 (**B**), d6 (**C**), and d8 (**D**). Splenocytes were stained for CD8, CD4, CD11b, CD19, F4/80, and NK1.1 and analyzed by flow cytometry. On the *γ*-axis, the absolute cell number of each population is shown. Values represent mean ± SD (*n* = 5–12 per group). **A** and **B** were analyzed by individual ordinary one-way ANOVA or Kruskal–Wallis test with multiple corrections performed by Tukey or Dunn’s test, respectively. **C** and **D** were analyzed by unpaired *t* test or Mann–Whitney test. For each day analyzed, the data were pooled from two independent experiments. All values represent mean ± SD. *p < 0.05, **p < 0.01, ***p < 0.001, and ****p < 0.0001.

To further characterize the immune response after CB-5083 administration in LCMV-infected mice, the indicated immune cell populations were analyzed in the blood (Fig. 3) and spleen (Fig. 4) on d2, d4, d6, and d8 post-infection by flow cytometry. To avoid suffering and death of mice, CB-5083 was only administered on d0 and d2 post-infection. For most analyzed cell types, barely any changes could be observed. Strikingly, CD8^+^ T cells were strongly reduced on d6 and d8 in the blood and d6 in the spleen of CB-5083-treated LCMV-infected mice (Figs. 3C, D and 4D), indicating that the expansion of CD8^+^ T cells is suppressed in these mice.

### Effect of VCP/p97 inhibition on cytokine levels in LCMV-infected mice

**Fig. 5:**
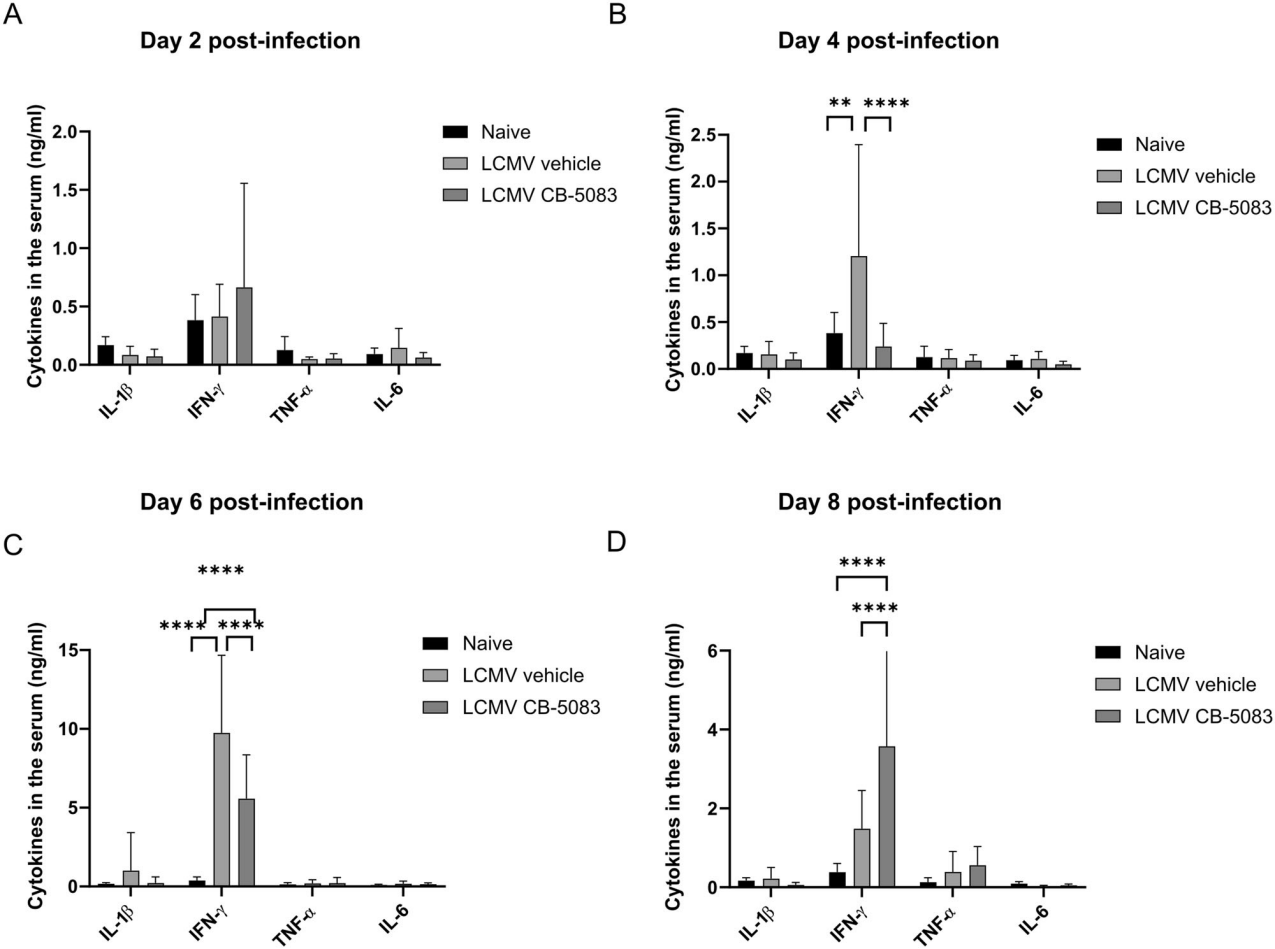
Cytokine in the serum of LCMV-infected CB-5083-treated mice. C57BL/6 mice were infected with LCMV (d0) and treated with 50 mg/kg CB-5083 or vehicle on d0 and d2. Blood was collected on d2 (**A**), d4 (**B**), d6 (**C**), and d8 (**D**). Serum was analyzed for IL-1β, IFN-γ, TNF-α, and IL-6 by ELISA. Values represent mean ± SD (*n* = 7–12). Data were analyzed by a two-way ANOVA followed by Tukey post hoc test. For each day analyzed the data was pooled from two independent experiments. All values represent mean ± SD. **p < 0.01 and ****p < 0.0001.

Plasma cytokine levels in patients suffering from IBMPFD are significantly perturbed [38]. Particularly, increased levels of epidermal growth factor and TNF-α were detected, which in excess, can cause muscle atrophy [39]. IL-1β, TNF-α, IFN-γ, and IL-6 plasma levels were measured on d2, d4, d6, and d8 post-infection. (Fig. 5). Even though we observed no alteration of IL-1β, TNF-α, and IL-6 serum levels, CB-5083 treatment affected the levels of IFN-γ in the serum. The reduction of IFN-γ in the serum in CB-5083-treated mice on d4 and d6 (Fig. 5B, C) correlates with a decreased number of CD8^+^ T cells, the main producers of IFN-γ after LCMV infection, in the blood and the spleen (Figs. 3 and 4). However, while IFN-γ serum levels decreased in vehicle-treated mice on d8 to levels observed on d4, IFN-γ serum levels in CB-5083-treated mice remained elevated (Fig. 5D).

### VCP/p97 inhibition after the priming phase

**Fig. 6:**
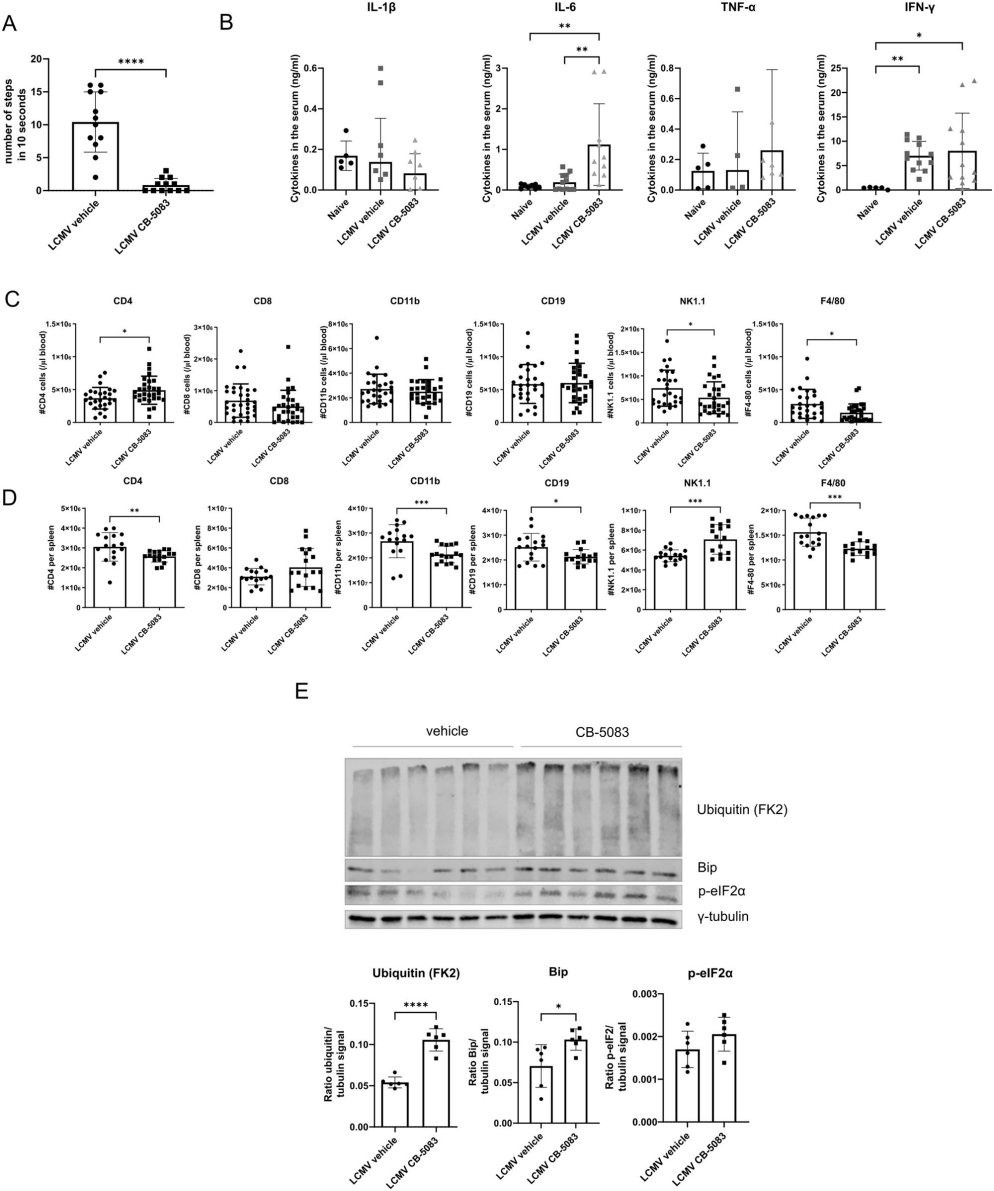
CB-5083 treatment of LCMV-infected mice after the priming phase strongly impairs health status. C57BL/6 mice were infected with LCMV and treated on d6 post-infection with 50 mg/kg CB-5083 or vehicle. Mice were analyzed 6 h post-CB-5083 treatment. **A** The movement of the mice was tracked and the individual numbers of steps in 1 min were counted. Values represent mean ± SD. Pooled data (*n* = 12) derived from two separate experiments were analyzed by unpaired *t* test. **B** IL-1β, IL-6, TNF-α, and IFN-γ concentrations were determined in the serum (*n* = 5–12). Values represent mean ± SD. For each analysis, a Kruskal–Wallis test followed by a Dunn’s test was performed. **C**, **D** The blood (**C**) (*n* = 27–32) and spleen (**D**) (*n* = 16-17) were collected and stained for CD4, CD8, CD11b, CD19, F4/80 and NK1.1 and analyzed by flow cytometry. The data was pooled from three different experiments and analyzed by unpaired *t* test. Values represent mean ± SD. **E** Splenocytes were lysed and analyzed via immunoblot for poly-ubiquitin accumulation, BiP, and p-eIF2α. On the *γ*-axis, the mean signal normalized to γ-tubulin ± SD from mice (*n* = 6) derived from two independent experiments analyzed by an unpaired *t* test is shown. All values represent mean ± SD. *p < 0.05, **p < 0.01, ***p < 0.001, and ****p < 0.0001.

Lymphocyte priming takes place during the first days of viral infection. So far, CB-5083 treatment has been performed during the priming phase (d0 and d2 post-infection), in which CB-5083 may affect antigen presentation. To investigate the effect of VCP/p97 inhibition at later stages of infection we started treatment on d6 post-infection. Unexpectedly, we observed a significant reduction in locomotion of the mice treated with CB-5083 compared to vehicle-treated mice within 6 h post-treatment (Fig. 6A). To avoid suffering of mice, 6 h post-CB-5083 treatment was defined as endpoint in this experimental set-up. Gross pathology of CB-5083-treated mice did not reveal any obvious cause for the observed toxicity (data not shown). Since a cytokine storm might explain toxicity within this short time period, we tested different cytokine levels in the serum (Fig. 6B). We observed a strong upregulation of IL-6 in infected mice treated with CB-5083. No alteration was detected for IFN-γ, IL-1β or TNF-α. Flow cytometry analysis of immune cells in the blood (Fig. 6C) and spleen (Fig. 6D) 6 h post-CB-5083 treatment did not reveal alterations in immune cell populations that could explain the observed toxicity of CB-5083. As described above, VCP/p97 inhibition leads to a disturbance of protein homeostasis in cells within a short time leading to an accumulation of ubiquitylated proteins (Supplementary Fig. 3). Indeed, we detected an accumulation of high-molecular ubiquitin forms in the spleen of CB-5083-treated mice 6 h post-treatment (Fig. 6E). Accumulation of ubiquitin is a hallmark of ER stress leading to induction of the UPR. Phosphorylated eukaryotic initiation factor-2α (p-eIF2α) and BiP (binding immunoglobulin protein aka GRP-78) are two components that initiate the UPR. Both proteins are slightly up-regulated in the spleen 6 h post CB-5083 treatment (Fig. 6E).

**Fig. 7:**
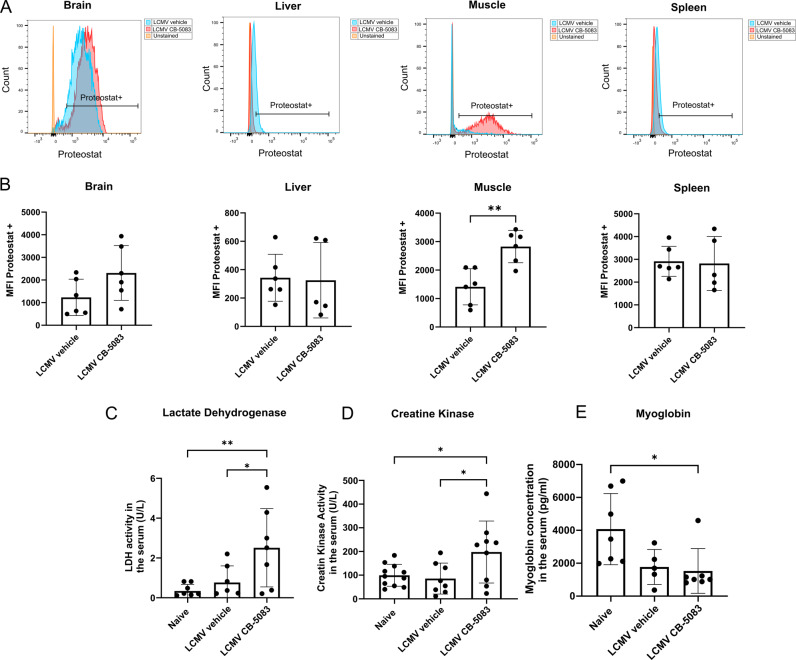
Muscular damage and aggregate formation. Mice were infected with LCMV (d0) and treated with 50 mg/kg CB-5083 or vehicle. The mice were analyzed 6 h post-CB-5083 treatment. **A**, **B** The brain, liver, quadriceps muscle, and spleen of mice were harvested, processed for flow cytometry analysis, and stained with proteostat. **A** The gating strategy is shown exemplarily. **B** Mean fluorescent intensity ± SD of proteostat^+^ cells of tissues from brain, liver, muscle and spleen is depicted on the γ-axis. Pooled data (*n* = 5–6) derived from two independent experiments was analyzed by unpaired *t* test. **C**–**E** Naive mice were used as controls. The serum of the mice (*n* = 5–11) was analyzed for LDH (**C**) and creatine kinase (**D**) with activity assay kits, and myoglobin (**E**) levels by ELISA. Each graph was analyzed via a one-way ANOVA followed by a Tukey post-hoc test. Data was pooled from two independent experiments. All values represent mean ± SD. *p < 0.05, **p < 0.01.

Aggresomes, structures of aggregated misfolded proteins within the cell, form when the capacity of the proteasome is exceeded [40]. Due to the essential role of VCP/p97 in proteasome function, we characterized the formation of aggresome-like structures in several organs, using the Proteostat® protein aggregation assay. We discovered that skeletal muscles in the mice treated with CB-5083 for 6 h depicted an increased formation of aggresome-like structures (Fig. 7B). No specific formation of aggresome-like structures could be detected in the brain, liver, or spleen. Furthermore, we measured serum levels of markers for tissue damage. We observed that the levels of lactate dehydrogenase (LDH) (Fig. 7C) and creatine kinase (CK) (Fig. 7D) were increased in the mice treated with CB-5083 compared to vehicle and naïve mice, whereas myoglobin levels were not altered (Fig. 7E). These data indicate that damage in muscular tissues is occurring shortly after CB-5083 treatment.

## Discussion

VCP/p97 is one of the most abundant proteins in eukaryotic cells. Inhibitors of VCP/p97 have shown efficacy in vitro and in two different xenograft acute myeloid leukemia models [41]. A phase I clinical trial with the VCP/p97 inhibitor CB-5083 in solid tumors and lymphoid hematological malignancies has been performed (NCT02243917/NCT02223598). CB-5083 was initially documented as clinically safe and well tolerated. However, due to off-target effects resulting in vision problems, which were later confirmed as temporal, the clinical development of CB-5083 was terminated due to suspected off-target activity against phosphodiesterase-6 (PDE6) [42]. CB-5339, an improved molecule developed by the same company, demonstrated its efficacy in multiple myeloma with minimal adverse events [43]. Furthermore, CB-5339 entered phase I for the treatment of AML and Myelodysplastic Syndrome (NCT04402541). Since it is a ubiquitously expressed protein there remains some concern regarding the general toxicity of targeting VCP/p97. Indeed, embryonic knockout of VCP/p97 in mice is lethal [44], whereas its mutations induce several neurological diseases [45]. Our study shows for the first time that inhibition of VCP/p97 increases the susceptibility to a viral infection in a CD8-independent manner.

The ATPase activity of VCP/p97 is essential for many cellular processes, including membrane-trafficking of proteins and Golgi reassembly after mitosis [46]. Hence, VCP/p97 has an essential role at multiple steps of viral infection and replication, like in retro-translocation from the ER lumen to the cytosol [47, 48] and unfolding [49]. CB-5083 treatment leads to accumulation of poly-ubiquitinated proteins within a short time (Supplementary Fig. 3) [21]. LCMV infection upregulates the XBP1s branch of the UPR (Fig. 2). Previously, Pasqual et al. showed that in vitro LCMV infection led to ATF6 upregulation [50]. However, we could not detect any upregulation of the ATF6 branch upon LCMV infection. Furthermore, we observed that simultaneous treatment with CB-5083 upregulated the XBP1 branch in a time-dependent way (Fig. 2). Xbp1s mRNA has been identified as a hallmark in many conditions characterized by the formation of insoluble intracellular aggregates such as Alzheimer’s [51], Huntington’s [52], and metaphyseal chondrodysplasia-type Schmid [53] diseases. IRE1α and its downstream target, XBP1, are activated in the skeletal muscle of mice upon injury [54], and this branch is essential for alleviating protein aggregation related to skeletal disease [55].

The role of VCP/p97 inhibition in LCMV infection in vivo in mice has not been investigated so far. Interestingly, mice were unable to cope with LCMV infection after VCP/p97 inhibition, resulting in increased viral titers on d8 post-infection (Fig. 1C). No alteration of viral titers was encountered in the first stages of infection (d2, d4, d6), which suggests that neither the innate immune response nor the viral replication were affected. Viral clearance in mice infected with LCMV-WE is strictly depended on CTLs. Indeed, the increased viral load in CB-5083-treated mice on d8 post-infection was associated with a strongly impaired LCMV-specific CTL response in these mice (Fig. 1B). Since the CTL response to all analysed T cell epitopes is reduced in CB-5083-treated mice, an effect of VCP/p97 on antigen presentation seems rather unlikely in this set-up. The reduced LCMV-specific CTL response is in line with a reduction of the CTL numbers in the blood on d6 and d8 post-infection (Fig. 3C, D). Lisiero et al. found that the activated CD8^+^ T cells in Nfkbia^NES/NES^ mice infected with LCMV were significantly reduced [56], suggesting that nuclear export of IκBα is crucial for anti-viral CTL responses. Whether reduced activation of NF-κB, as measured by IκB degradation, in CB-5083-treated cells (Fig. 2B) contributes to the reduced CTL response at later days remains elusive. VCP/p97 likely plays a major role in mitochondrial maintenance [57]. Inactivation of VCP/p97 in neuronal cells resulted in loss of mitochondrial membrane potential and increased production of reactive oxygen species (ROS) [58]. Interestingly, elevated ROS levels in granulocytes decreased survival of LCMV-specific CTLs [59]. Hence, the reduced LCMV-specific CTL response observed in CB-5083-treated mice, might derive from elevated ROS levels in these mice.

A single dose of CB-5083 in mice after the T cell priming and initial expansion phase on d6 post-infection led to a drastic drop in the health status within hours, indicated by a strongly reduced motility of the mice (Fig. 6A). Analysis of several immune cell populations in these mice did not reveal excessive cell death within the short 6 h treatment (Fig. 6C, D). The observed toxicity of CB-5083 in these mice might be linked to the formation of aggresome-like structures in skeletal muscle (Fig. 7B) and increased IL-6 secretion in the serum (Fig. 6B). VCP/p97 has an essential role in lysosomal homeostasis, that, when dysregulated, leads to myofiber necrosis [60]. This turns VCP/p97 into an essential element in muscle homeostasis.

To check whether an IBMPFG-like syndrome, which is characterized by progressive myopathy, was responsible for the observed rapid drop in health status, we measured some markers of rhabdomyolysis in the serum. We detected an alteration in the LDH levels (Fig. 7C), which reflects non-specific organ damage and can be a consequence of muscle conditions, hepatocyte damage, or hemolysis [61]. Interestingly, in patients suffering from IBMPFD, the serum CK levels are normal or slightly increased whereas protein aggregation in the muscle fibers is a typical histological feature [62]. CB-5083 leads to an accumulation of poly-ubiquitylated proteins (Supplementary Fig. 3), which induces the UPR. Indeed, in LCMV-infected CB-5083-treated cells the UPR was upregulated to a higher level, compared to uninfected cells (Fig. 2). This is in agreement with the increased protein aggregation observed in muscle fibers in CB-5083-treated LCMV-infected mice (Fig. 7B). Likewise, we found slightly higher levels of CK in the serum of mice treated with CB-5083 (Fig. 7D), which were significantly higher compared to both naïve and vehicle-treated mice. This might indicate increased muscle damage in virus-infected mice with inhibited VCP/p97 activity. However, we could not detect an alteration in the myoglobin levels (Fig. 7E), which is an iron- and oxygen-binding protein expressed in cardiomyocytes and oxidative skeletal (low) muscle fibers of vertebrates [63]. Remarkably, in this study, we analyzed the protein aggregation in the quadriceps muscle of the mice, which is composed of nearly 100% of the fast-twitch type [64]. Whether fast-type muscles are more sensitive to VCP/p97 inhibition is currently unknown.

Similar to its clinical trial in cancer, we observed increased toxicity of CB-5083 in virus-infected mice. Even though the intrinsic mechanisms for the observed toxicity in our study could not be conclusively clarified, we suggest an overlap of different pathomechanisms in virus-infected mice leading to toxic protein aggregation and thus to cell death. The data obtained in this study has important implications for the clinical use of VCP/p97 inhibitors. Our results suggest careful monitoring and considering antiviral prophylaxis of VCP/p97-inhibitor-treated patients.

## Supplementary information

Supplemental Material

aj-checklist

## Data Availability

The data that support the findings of this study are presented in the Supporting Information. Further data are available from the corresponding author upon reasonable request.
