# Supplementary material for: Valosin-containing protein (VCP/p97) inhibition reduces viral clearance and induces toxicity associated with muscular damage
Source: Cell Death Dis. 2022 Dec 1;13(12):1015. doi: 10.1038/s41419-022-05461-w (PMC9715549; doi:10.1038/s41419-022-05461-w)
Supplement: Supplementary file 1 — Supplemental Material [file 41419_2022_5461_MOESM1_ESM.pdf]

Supplementary Data

Valosin-containing protein (VCP/p97) inhibition reduces viral clearance and induces toxicity associated with muscular damage.

Marta del Rio Oliva[1] and Michael Basler[1, 2]

[1] Division of Immunology, Department of Biology, University of Konstanz, Konstanz, Germany

[2] Biotechnology Institute Thurgau at the University of Konstanz, Kreuzlingen, Switzerland

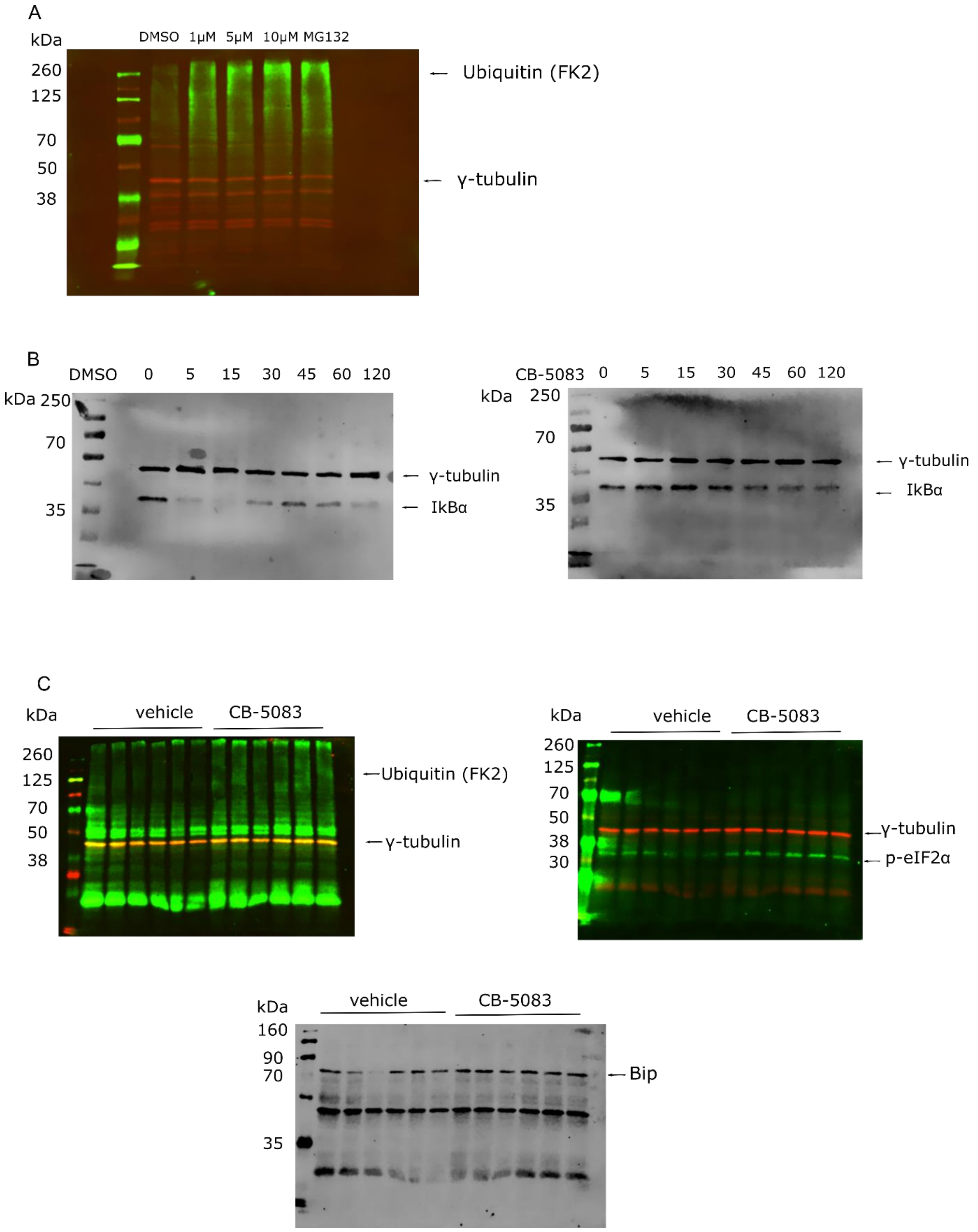

Supplementary Figure 1. Uncropped immuoblots: (A) Uncropped immunoblot from Figure 1A against Ubiquitin (FK2) and γ-tubulin. (B) Uncropped immunoblot from Figure 2B depicting IkBα and γ-tubulin. (C) Uncropped immunoblots from Figure 6E showing Ubiquitin (FK2), γ-tubulin and p-eIF2α in DMSO and CB-5083-treated cells.

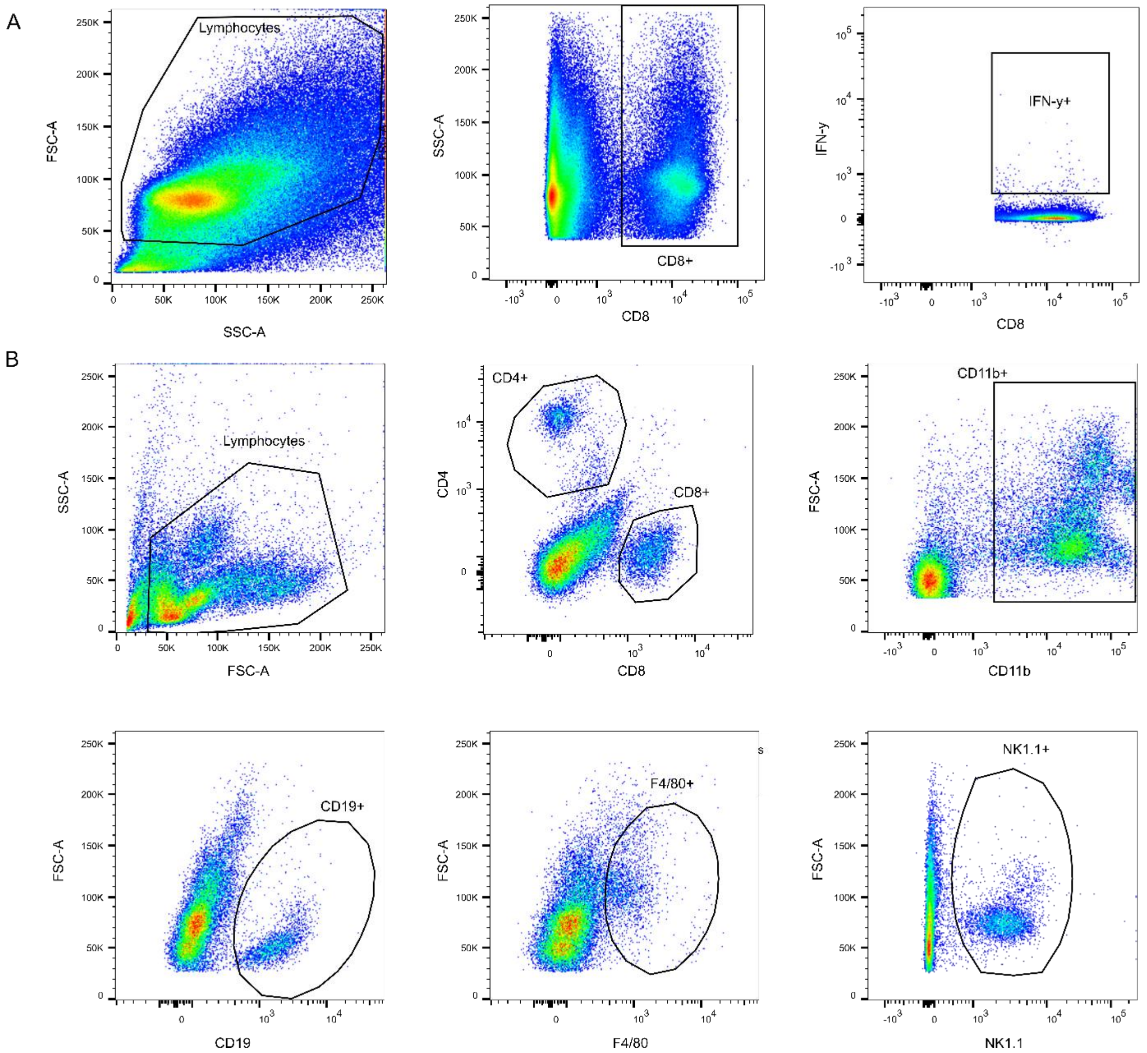

Supplementary Figure 2: (A) Gating strategy for flow cytometry in Figure 1B, in which C57BL/6 mice were infected with LCMV and treated with 50 mg/kg CB-5083 or vehicle on d0 and d2 post-infection. Spleens were harvested on d8 post-infection, left unstimulated or stimulated *in vitro* with LCMV peptides for 5h, and analyzed by flow cytometry after staining for CD8 and intracellular IFN-γ. (B) Gating strategy for Figure 3 and Figure 4, in which C57BL/6 mice were infected with LCMV and treated with CB-5083 or vehicle on d0 and d2 post-infection. Blood and splenocytes were collected on d2, d4, d6 and d8 post-infection, stained for CD8, CD4, CD11b, CD19, F4/80, and NK1.1 and analyzed by flow cytometry.

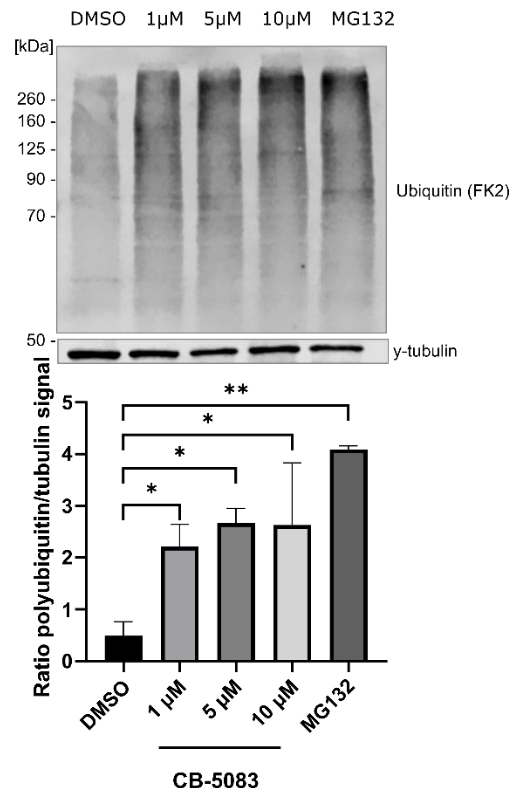

Supplementary Figure 3: MC57 cells were treated *in vitro* with 1, 5, or 10 µM of CB-5083 for 6h. The proteasome inhibitor MG132 was used as positive control. The lysates were analyzed via immunoblot for poly-ubiquitin accumulation. The quantification of the signal is depicted normalized to γ-tubulin. Data was pooled from two independent experiments and analyzed by One-way Anova followed by a Fisher´s LSD test. All values represent mean ± SD *$p<0.05$, **$p<0.01$.

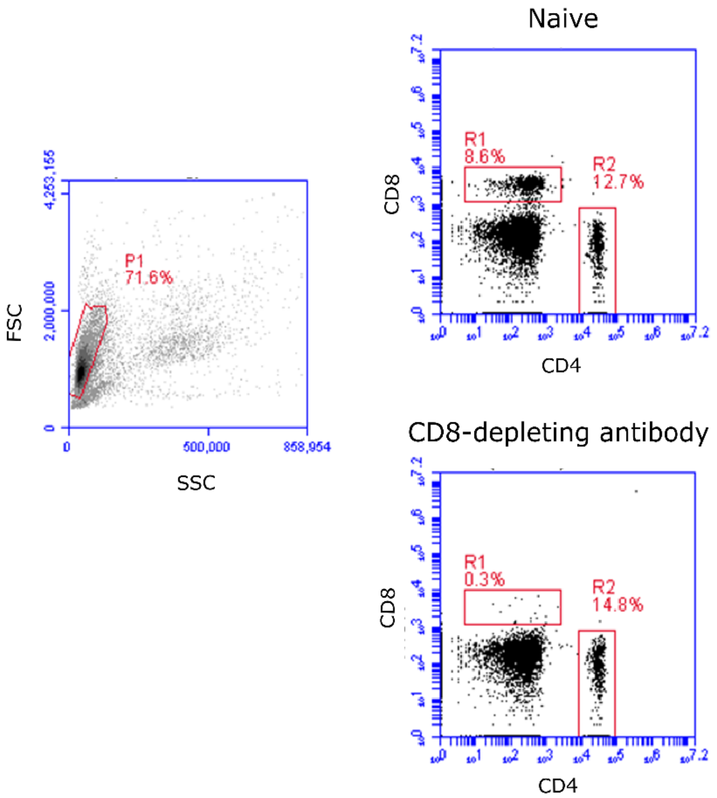

Supplementary Figure 4: Flow cytometry gating strategy of the blood of mice treated with CD8-depleting antibody. C57BL/6 mice were treated i.p with a CD8-depleting antibody. Three days later, the mice were infected with LCMV and treated daily with 50 mg/kg CB-5083. The CD8-depleting antibody was re-administered on day 2 post-infection. The absence of $CD8^+$ cells was confirmed by staining blood samples with α-CD8 and α-CD4 antibodies on day 1 post-infection.

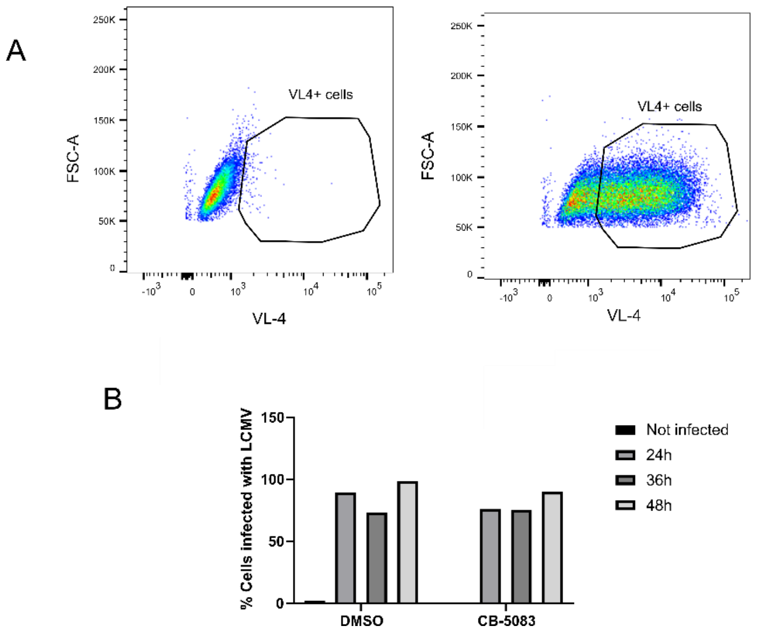

Supplementary Figure 5: Flow cytometry gating strategy and quantification of MC57 cells infected with LCMV after 24h post-infection. Cells were stained with anti-LCMV-NP antibody (VL-4) (28) and secondary anti-rat Ig-FITC (BD Pharmingen). All values represent mean ± SD. The experiment was repeated twice, yielding similar results.

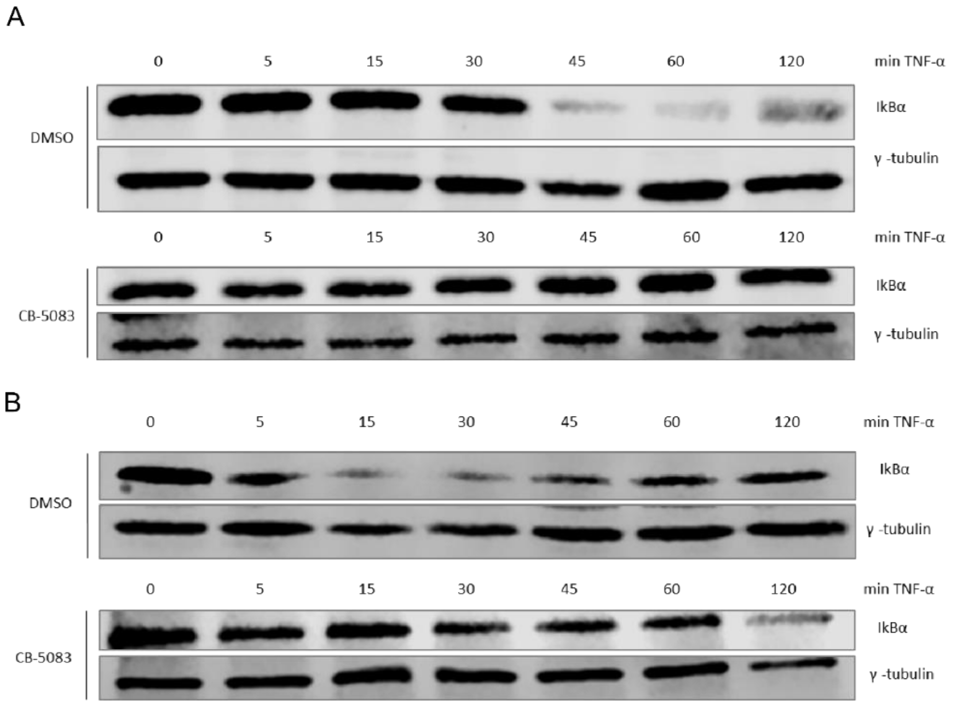

Supplementary Figure 6. Cytokine-induced IκB-α degradation in B8 (A) and T1 (B) cells. The cells were incubated with DMSO or 5 µM CB-5083 for 6h. Then, the cells were stimulated with 200 U/ml TNF-α and harvested at indicated time points. Samples were lysed and an SDS-PAGE and immunoblot for IκBα were performed. γ-tubulin was used as a loading control. The experiment was repeated twice yielding similar results.
